# Off-Axis Illumination and Epicollection Confocal Brillouin Scattering Microspectroscopy

**DOI:** 10.1021/acs.analchem.5c06290

**Published:** 2026-02-02

**Authors:** Hiroharu Yui, Yuui Fujiyama, Shu-hei Urashima, Takeru Ota, Hiroshi Hibino

**Affiliations:** † Department of Chemistry, Faculty of Science, 26413 Tokyo University of Science, 1-3 Kagurazaka, Shinjuku, Tokyo 162-8601, Japan; ‡ Department of Pharmacology, Graduate School of Medicine, The University of Osaka, 2-2 Yamadaoka, Suita, Osaka 565-0871, Japan; § Global Center for Medical Engineering and Informatics, The University of Osaka, 2-2 Yamadaoka, Suita, Osaka 565-0871, Japan

## Abstract

Viscoelastic properties of tissues and biologically relevant fluids therein are important to maintaining their structures and functions. Confocal Brillouin scattering microspectroscopy (μCBS) is a promising tool to address such local viscoelastic properties in nonlabeling and noninvasive manners. However, since the intensity of Brillouin scattering is extremely weak, the strong background by reflected light and Rayleigh scattering often hinders the quantitative analyses of viscoelastic properties in buried tissues and biological fluid. Here, we report an optical configuration for the reduction of such strong backgrounds based on the combination of off-axis illumination and epicollection for μCBS. As a test sample, aqueous solutions of bovine serum albumin (BSA) with relevant electrolytes were measured through thick glass windows (1.25 mm) of quartz cuvettes. Such thick windows act as strong reflectors and scatterers for the focused illumination light due to interfacial reflections and multiple reflections therein. The series of Brillouin scattering spectra of BSA solutions up to 20 wt % buried under the thick window were successfully analyzed owing to the effective reduction of those strong backgrounds. Further, it was clarified that hydrated water molecules surrounding BSA non-negligibly contribute to Brillouin scattering bandwidth, indicating that it would be also applicable to detect conformational changes of proteins in fluids. The off-axis illumination and epicollection optical configuration would further enhance the μCBS’s ability for its future application in biochemistry and biomedicine to assess the viscoelastic properties of tissues with complex structures and biologically relevant fluids, such as blood and lymph flowing therein.

## Graphical abstract

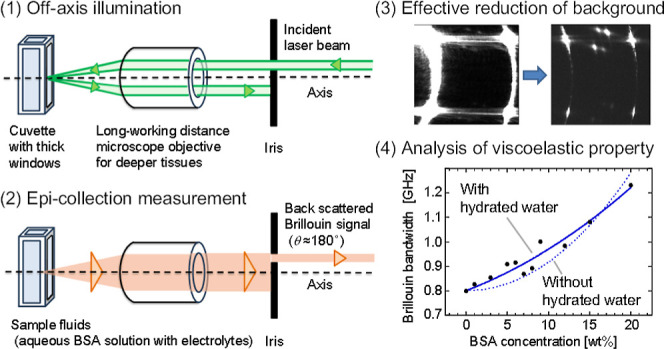

## Introduction

Since the theoretical prediction of phonon–photon scattering by Leon Brillouin in 1922, Brillouin scattering has been proposed and applied as a spectroscopic analytical tool to investigate local thermodynamic and mechanical properties in a nonlabeling and noninvasive manner.^1^ In the last two decades, by applying narrow-band laser technologies as a light source and a Fabry–Pérot interferometer (FPI) and a virtually imaged phased array (VIPA) as a spectrometer, contrast images of the stiffness in cells and tissues have been reported through the change in Brillouin scattering frequency.^1–5^ Further, by combining a confocal microscope with Brillouin spectroscopy (μCBS), three-dimensional mechanical imaging has been realized.^6^ Such imaging technology of the mechanical properties in three-dimensionally inhomogeneous tissues would provide a promising modality for investigating the mechanism of their biological function and pathogenesis.

However, some difficulties still lie in evaluating such mechanical properties from the Brillouin scattering spectra. One of the non-negligible difficulties is the strong background due to the simultaneously induced Rayleigh scattering. Strong Rayleigh scattering from the interfaces of complexed tissue structures with different indices often hinders quite weak Brillouin scattering signals. Further, the reflected stray lights from the multiple optical components in the microscope objective and in the instrument also induce non-negligible background signals. To extend the applicability of μCBS to deeper and buried tissues for biological and medical purposes, further techniques for the effective reduction of the strong background have been required to overcome such inherent difficulties in microscopic measurements on inhomogeneous biological samples.

To overcome such inherent difficulties of μCBS, here, we report the combined configuration of an off-axis illumination and an epicollection for microspectroscopy to further reduce the strong backgrounds induced by Rayleigh scatterings and stray reflected lights. We applied the off-axis illumination and epicollection technique to aqueous solutions of bovine serum albumin (BSA) up to 20 wt % with relevant electrolytes as a model biological fluid. The samples were filled in a quartz cuvette with thick windows acting as strong reflectors and scatterers of the focused illumination beam by microscope objectives. The Brillouin scattering spectroscopic data of the BSA solutions were successfully obtained, and the non-negligible contribution from the hydrated water molecules of the albumin to the viscous properties of albumin solutions was detected and analyzed based on a biphasic model for viscoelastic systems.

## Experimental Methods

### The Optical Setup of Confocal Brillouin Scattering Microspectroscopy

The optical diagram of the confocal Brillouin microspectroscopy constructed here is illustrated in Figure 1. The representative optical devices and configurations for confocal Brillouin microspectroscopy reported previously such as multistage VIPA,^7^ Lyot stop,^8^ and utilizing calibration liquid^9^ have been applied in the instrument presented here. The light source was a single-frequency DPSS laser (wavelength: 532 nm, intensity max: 300 mW, spectral line width: <1 MHz, and polarization purity: >100:1, Samba, Cobolt). The power of the illumination laser beam was controlled with a neutral density filter wheel (NDC-50C-2M-A, Thorlabs) to avoid excess heating of the sample solutions. The diameter of the laser beam was expanded and collimated with a lens pair (focusing length; L1: 50 mm, L2: 500 mm). Then, only the center area of the laser beam was passed through the pinhole (Ph1) with a diameter of 2 mm. To preserve the polarization purity of the laser, a nonpolarizing prism beam splitter (reflection: 50%; transmission: 50%, BS013, Thorlabs) was utilized to split the excitation beam into two for a sample line and a reference one. The same microscope objectives with a long-working distance (working distance (WD): 20 mm, focusing length: 10 mm, and numerical aperture (NA): 0.40, M Plan Apo NIR20x, Mitsutoyo) were used for both the sample and reference lines. The reason for the application of long-WD microscope objectives is for the future application toward optical coherent tomography (OCT) to investigate the local viscoelastic properties of buried tissues with remarkable thickness with mm to cm scales and biorelevant fluids such as blood and lymph flowing therein.^10,11^ The choice of the numerical aperture (NA) of the microscope objective is also important for the analysis of the Brillouin scattering data. This is because the frequency shift of the Brillouin scattering (ν_B_) depends on the angle (θ) between the incident and scattered light, given by

1 $$\nu_{B}=\left(2n/\lambda_{0}\right)V\;\sin\left(\theta /2\right)$$

where *n* is the refractive index of the sample material in the scattering volume, λ_0_ is the wavelength of the incident laser light (here: 532 nm), and *V* is the sound velocity of the sample medium.^1–6^ The NA of the microscope objective becomes wider, it collects a wider angle of the scattering light, resulting in the apparent broadening of the line width of the Brillouin scattering spectra. Preceding theoretical and experimental investigation on the relation between the NA and the frequency shift and the line width of Brillouin scattering, the choice of the NA below 0.3 and the backscattered (θ = 180°) observation minimize the unwanted frequency displacement and the apparent broadening of the line width of the Brillouin scattering spectra.^12–17^ Here, we utilized microscope objectives with NA = 0.4 with a pupil lens diameter of 8.0 mm. However, we reduced the diameter of the incident laser beam to 2.0 mm (1/4 of the pupil lens diameter) by iris 1 in Figure 2, the effective NA was reduced to 0.1, which contributes to avoid unwanted displacement in the scattering frequency and apparent broadening in the line width of the Brillouin scattering spectra. The expected effect on spectral broadening Δν_B_ (= ν_B max_ – ν_B min_) under the effective NA (=0.1) and epidirection measurement (θ_center_ = 180°) conditions was estimated as follows. By assuming the refractive index *n* = 1.4 and the sound velocity *V* = 1500 [m/s] as a typical value of aqueous semidilute protein solution and by combining eq 1 and NA = *n* sin θ, Δν_B_ can be estimated as ca. ±0.0026 [GHz]. Although the range of θ has a finite value of ca. ±2° even for the effective NA = 0.1, the spectral broadening effect was minimized owing to the epidirection (θ = 180°) measurement (i.e., the robustness of the θ dependence of eq 1 at around θ = 180°). This value is effectively low for the analysis on the dependence of the Brillouin scattering frequency shift and the line width by varying the concentration of the protein solutions presented here. The backscattered (θ = 180°) Brillouin scattering lights from the sample (distilled water and albumin aqueous solutions with electrolytes of typical biological conditions) and the reference (pure methanol) were collected by each objective lens and back to the beam splitter. Then, they were passed through the same pinhole (ϕ = 50 μm) in the confocal configuration and collimated by the lens pair (focusing length; L3: 100 mm, L4: 50 mm). To avoid excess heating and unwanted increase in local temperature of the sample liquids, a mechanical shutter of the excitation laser beams for both the sample and reference was basically closed and was opened only when the Brillouin scattering measurements were carried out.

**Figure 1:**
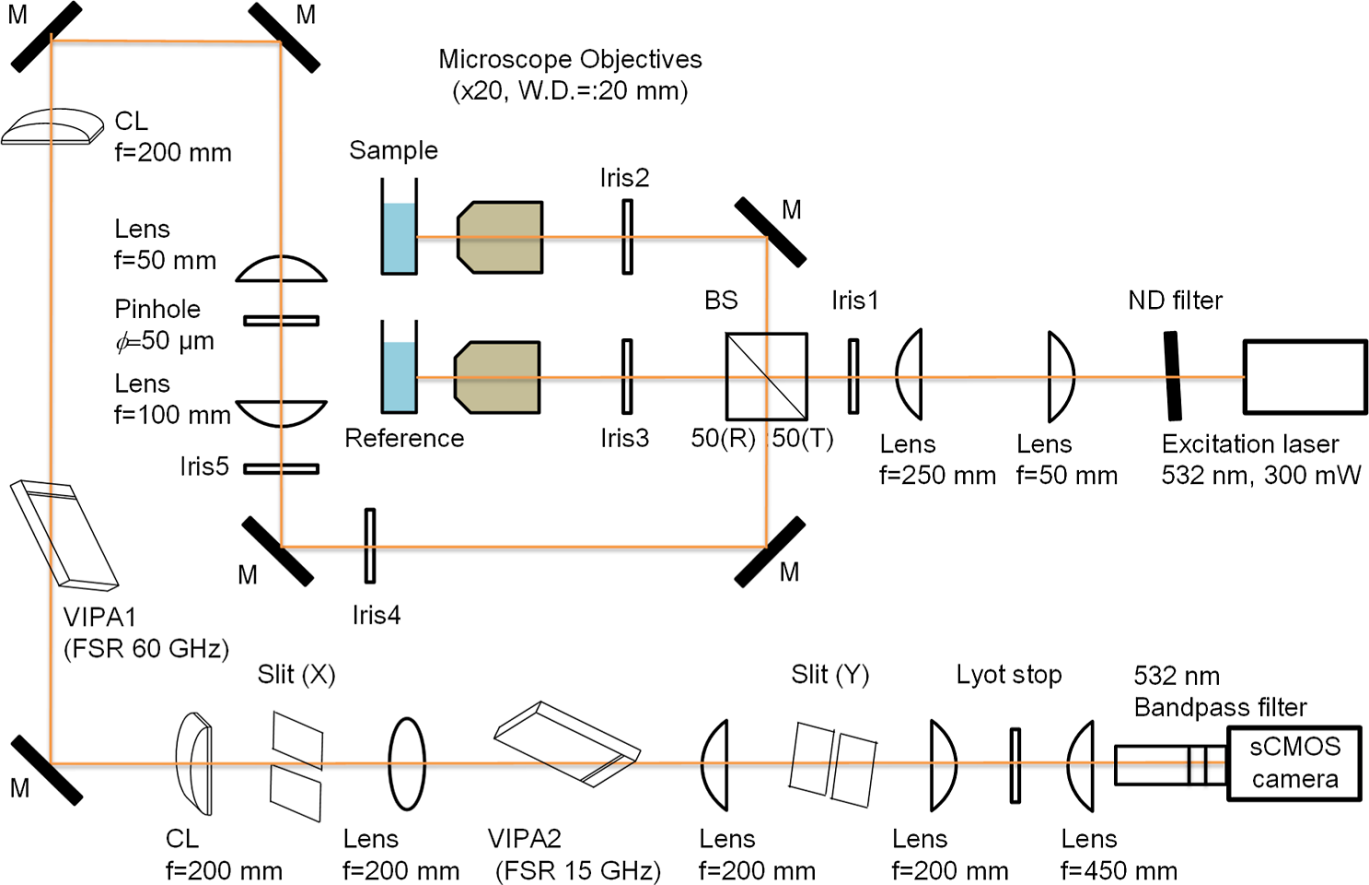
Setup for the confocal Brillouin microscopy with a reference line. The laser beam was split into two with a nonpolarizing beam splitter (reflection: 50%: transmission: 50%) for a sample line and a reference one. Brillouin scattering lights from the both sample and reference lines are collected in the epidirection with each microscope objective. These Brillouin scattering lights passed through the confocal pinhole (diameter: 50 μm) and tandem VIPAs with different resolutions (FSR 60 GHz in the horizontal axis and FSR 15 GHz in the vertical axis toward the optical bench used here). Reflected Rayleigh scattering that was not used for the analysis was rejected with the slits *X* and *Y*. After passing through a Lyot stop and a bandpass filter of 532 nm, the Brillouin scattering signals from the sample and reference lines were focused onto the CCD sensor in the sCMOS camera and recorded simultaneously.

**Figure 2:**
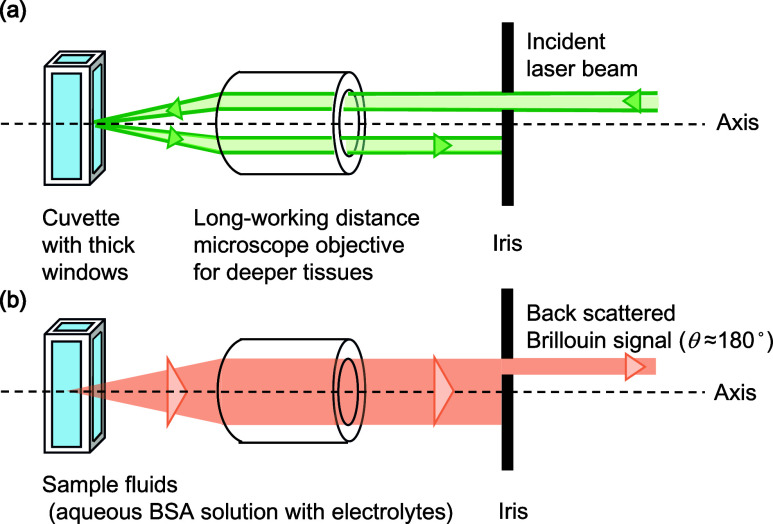
Optical configuration of an off-axis illumination and epicollection for the confocal Brillouin microscopy constructed here. (a) The laser beam was introduced in parallel but with an off-axis line to the center of the microscope objectives. (b) Backscattered light (scattering angle: around 180° to the illumination light) was collected with the same objective lens in the same optical pass of the illumination light. Unwanted reflected and stray lights were spatially blocked with an iris placed behind the objective lens.

The spatially filtered and collimated signals from the sample and reference were introduced to a tandem VIPA spectrometer. The spectrometer consists of two cylindrical lenses (C1: 200 mm; C2: 200 mm) and two VIPA etalons (VIPA1: OP-6721-1686-2, 60 GHz FSR, VIPA2: OP-6721-6743-2, 15 GHz FSR, Light Machinery). Two *X* and *Y* slits were used to select dispersion orders by the VIPA etalons and to reject other orders and unwanted stray lights. Further, a Lyot stop was also implemented in the spectrometer to reduce diffraction noise patterns of Rayleigh scatterings as mentioned above.^8,18^ The Lyot stop would be beneficial when the Brillouin scattering signals are acquired from the interfacial region between two different materials (such as biological tissues and fluids) with different indices. The transmitted light was focused onto a sCMOS sensor camera (NEO-5.5-CL3, Andor Technology) with each pixel size being 6.5 μm, corresponding to the 0.07 GHz/pixel in the 15 GHz direction of the sCMOS image under the present condition with focusing length (450 mm) of the lens in front of the sCMOS camera (Figure 1). This value is considered to be enough to discuss the frequency shift and line width with sub-0.1 GHz resolution by curve-fitting analysis of the concertation dependence of the albumin aqueous solutions. If one wants to discuss further precise shift and line width for other purposes, one can control the focused image size on the sensor of the sCMOS camera by varying the focusing length of the lens in front of the sCMOS camera.

### Expected Reduction of the Spatial Resolution and the Optical Throughput for an Off-Axis Illumination

Compared with the fulfilled illumination of the pupil lens of the optical microscope objective, the reduction effect of the spatial resolution with the off-axis illumination should be estimated at first. The confocal length *l*_0_ (axial resolution) and diameter *d*_0_ (lateral resolution) can be estimated from the following eqs 2 and 3:^19^

2 $$l_{0}=\left(\frac{\pi }{6}\right)\frac{{d_{0}}^{2}}{\lambda_{0}}$$

3 $$d_{0}=\left(\frac{4}{\pi }\right)\frac{f\lambda_{0}}{d}$$

where λ_0_ is the excitation wavelength (532 nm) and *d*_0_ is the incident signal diameter. The present condition (illumination diameter: 2 mm) of the focusing length (*f*) of the objective lens is 10 mm, and the corresponding *d*_0_ and *l*_0_ for the off-axis illumination were calculated to be 3.39 and 11.3 μm, respectively. If we assume the typical on-axis configuration with fulfilled illumination for the same microscope objective (NA = 0.4, pupil lens diameter: 8 mm), expected *d*_0_ and *l*_0_ of the focal volume can be reduced to be 0.85 and 0.71 μm, respectively. Here, we reduced the diameter of the illumination beam to be 2 mm to minimize the unwanted spectral broadening in the Brillouin line width. In contrast, if one prefers higher optical throughput (for a fast imaging) and/or spatial resolution (for a precise imaging), one can maximize the diameter of the off-axis illumination beam to be the half of that in an on-axis and fulfilled illumination (in the present case, 4 mm can be applied). In this case, the expected *d*_0_ and *l*_0_ are 1.69 and 2.82 μm, respectively. Thus, we can roughly estimate the reduction of the spatial resolution for the imaging purpose to be 1/2 in the lateral direction and 1/4 in the depth direction under off-axis illumination compared to those on-axis one under the same microscope objective. Under this condition, optical throughput becomes 1/4 (the square of the beam diameter) and the spatial resolution is 1/8 (the product of the enlarged focal lengths of lateral and depth directions) compared to those in the on-axis configuration with fulfilled illumination of the pupil lens of the same microscope objective. In general, the NA of the microscope objective is the most important parameter for the spatial resolution for typical on-axis and fulfilled illumination. In addition, when one applies the off-axis illumination for imaging purpose, the diameters of the illumination beam and the pupil lens of the microscope objective are also important parameters to satisfy the aimed spatial resolution under off-axis illumination.

### The Roles of the Reference Line Constructed in Our Instrument

It is worth noting that the signal and reference were simultaneously measured and recorded in the same image. This would contribute to the reduction of the unwanted effect of the frequency fluctuation of the Brillouin scattering signal due to the slight changes of the temperature in the laser system and of the sample under long-time operation. Owing to the simultaneous measurement and recording of the reference signal, we are able to monitor the stability of the laser system and also to calibrate the frequency and the line width by the “reference” signal simultaneously recorded in the same sCMOS image for further quantitative analysis afterward. This is because the Brillouin frequency is sensitive to the slight change in the temperature of the laser system that sometimes induces non-negligible affection on the accuracy of the readout of the Brillouin shift and also the line width. If it is needed, the simultaneously recorded reference signal can also be utilized to calibrate the unwanted frequency shift that occurred by the temperature fluctuation of the laser under long-time operation. As for the analysis of Brillouin line width, the reduction of the effective NA with off-axis illumination would favorably contribute to the reduction of the unwanted broadening of the Brillouin bandwidth. In the present tandem configuration, if we want to know the natural value of the sample line width Γ_B_ (sample_natural) to evaluate the absolute values of its viscosity, we only have to calculate the ratio of the measured sample/reference line widths Γ_B_ (sample_measured)/Γ_B_ (reference_measured) and then calculate the product of the ratio with the natural Brillouin bandwidth Γ_B_ (reference_natural) of the reference that has been already experimentally measured and/or theoretically calculated according to the following eq 4:

4 $$\Gamma_{B}\left(sample\text{\_}natural\right)=\left(\Gamma_{B}\left(sample\text{\_}measured\right)/\Gamma_{B}\left(reference\text{\_}measured\right)\right)\cdot \Gamma_{B}\left(reference\text{\_}natural\right)$$

Another important reason for the instrumentation of the reference line is that it would help ensure the system stability to discuss the trend by varying the experimental conditions. Since the Brillouin measurements are sensitive to both the frequency fluctuation of the laser system and the observation angle (and range), deconvolution analysis with the instrumental responses from the measured experimental values is generally required. On the other hand, with the reference line constructed here, we are able to simultaneously monitor the stability of the laser system (especially in the frequency fluctuation and in the pointing stability) during the long-term operation such as for the monitoring the biochemical reactions and for wider range 3D imaging of relatively large tissues. If necessary, one can calibrate the frequency shift and the bandwidth by comparing them with the simultaneously recorded Brillouin spectra of the reference sample afterward.

### The Role of the Confocal System Constructed in Our Instrument

In the present experimental setup, the thick optical window (1.25 mm) of the quartz cuvettes strongly reflects the illumination lights at the air/quartz and sample/quartz interfaces. However, the off-axis illumination configuration applied here effectively avoids the unwanted strong reflected light from the front and backward interfaces of the optical window going back through the illumination line. These strong reflected lights are effectively blocked by iris 2, resulting in the effective reduction of the background signal. Only the Brillouin and Rayleigh scattering lights (θ = 180°) that went back to the axis of illumination beam can pass through iris 2. The same off-axis illumination and epicollection configuration are also applied to the reference line through iris 3. However, the inhomogeneous structures in tissue specimens and the various optical parts in the beamline may also be other origins of the unwanted reflected and stray light for the Brillouin scattering measurement. The confocal system with a pinhole (ϕ = 50 μm) shown in Figure 1 helps the selective passing of the signal light from the aimed focal volumes in the buried position of the sample. The present condition of the diameter of the signal after passing through iris 5 (2 mm) and the focusing length (*f*) of L3 in the confocal part is 100 mm, *d*_0_ at the beam waist and *l*_0_ in the confocal length were calculated to be 33.9 μm and 1.13 mm, respectively. The diameter of the confocal pinhole is 50 μm, and it is above the diameter of the beam waist (33.9 μm). Thus, it does not reduce the optical throughput. Further, as mentioned above, the focal volume in the sample by the microscope objective under off-axis illumination at the present conditions (diameter: 2 mm, focal length: 10 mm) was 3.39 μm in lateral and 11.3 μm in depth directions. Thus, the main role of the confocal system under the present conditions is for the cut of the unwanted reflected and stray light induced by the optics along with the beamlines for both sample and reference. If one wants to further improve the spatial resolution in both the lateral and depth directions by the optical pinhole, it would be recommended to use the same optical microscope objectives instead of the lens pair of the confocal system and to reduce the diameter of the pinhole for the aimed spatial resolution.

### Sample Preparation of Model Biorelevant Fluids

Aqueous solutions of bovine serum albumin (BSA) were prepared as a model sample for a biorelevant fluid. This is because albumin is an abundant and representative protein in the blood and lymph. For example, in the lymph of human bodies, the weight concentration of albumin is 4.1–5.1 g/dL (5.9–7.3 wt %) in that of whole protein (6.6–8.1 g/dL). Thus, to represent the protein wt % by albumin, we prepared the albumin aqueous solution with concentrations of 0, 1, 3, 5, 6, 7, 8, 9, 12, 15, and 20 wt %, including both lighter and heavier references. As a base water for preparing aqueous albumin solutions of BSA, distilled water (165-08245, FUJIFILM Wako Pure Chemical) and BSA (purity ≥ 98%, A2934,Sigma-Aldrich) were used for preparing the aqueous solution. The concentrations of the electrolytes (Na^+^, K^+^, and Ca^2+^) in the BSA aqueous solutions were set at 150 mM, 5 mM, and 1.2 mM, respectively, by adding NaCl, KCl, and CaCl_2_ salts (191-01665, 163-03545, 038-24985, FUJIFILM Wako Pure Chemical). The pH of the BSA aqueous solutions was adjusted to be 7.4 in the HEPES buffer solution (348-01372, FUJIFILM Wako Pure Chemical) by adding a dilute NaOH aqueous solution. The model fluids were loaded in quartz cuvettes with 1.25 mm-thick windows (F15-UV-10, GL Sciences).

## Results and Discussion

At first, the reduction of the stray lights as a background signal was examined by comparing the on- and off-axis illuminations (Figure 3). The effectiveness of the off-axis illumination was clearly observed in the reduction of stray light from both sample and reference arms. In general, since the difference in the brightness of the signals between the Rayleigh and Brillouin scattering is too large, the signal spots of Brillouin scattering are hardly recognized, especially for the observation of the strong scatterers and reflectors. The present optical configuration effectively blocked the reflected strong Rayleigh scattering signals at various interfaces; both Brillouin signals of water and methanol can be clearly observed at the sCMOS sensor. The appropriate balance in the intensity of the Rayleigh scattering and the Brillouin scattering signals helps us to calibrate the pixel number to the Brillouin shift.

**Figure 3:**
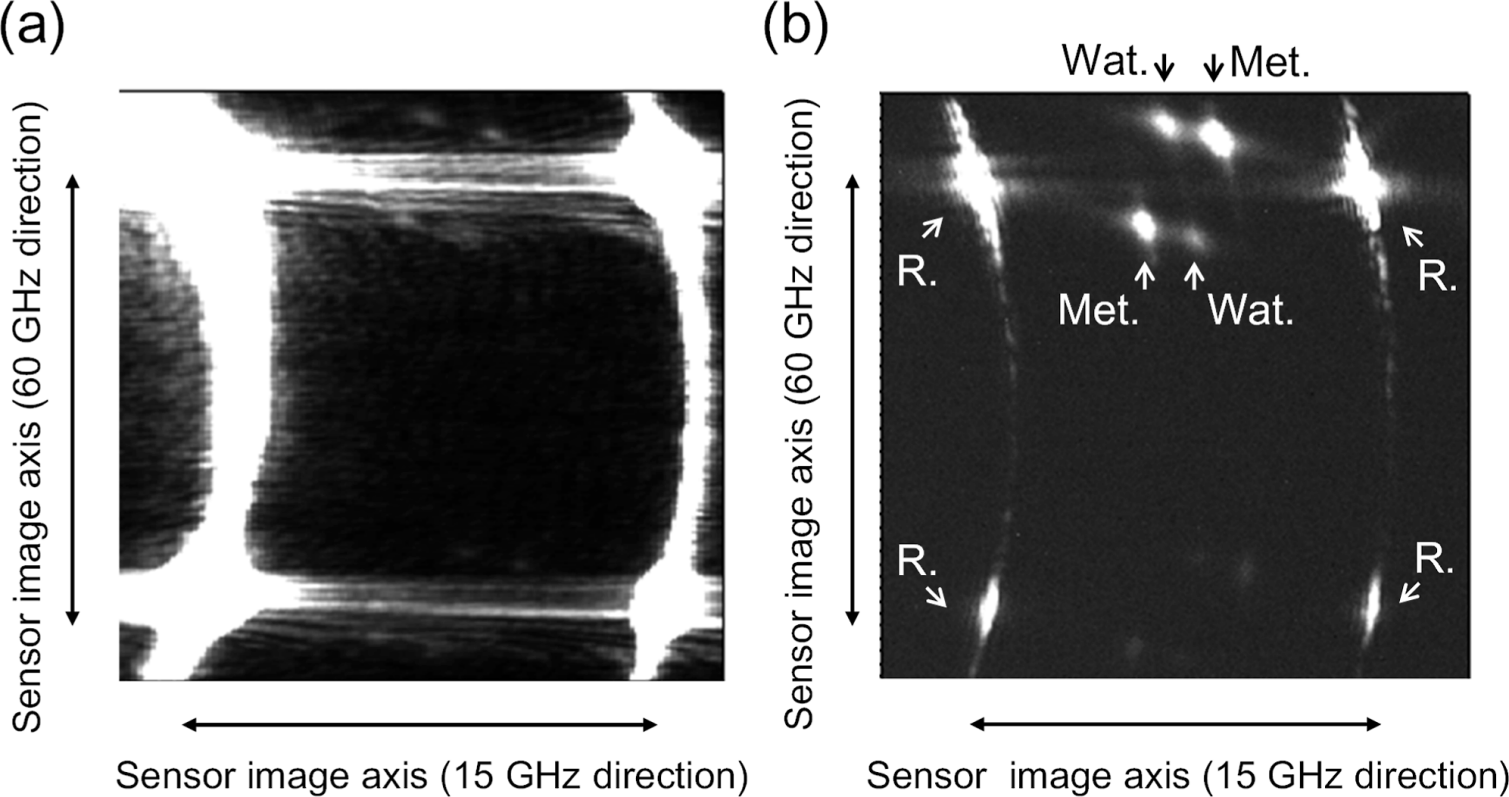
Comparison of the images of the sCMOS sensor between (a) the on-axis and (b) the off-axis illuminations. Each sCMOS image was acquired with 20 s accumulation. In the former, strong Rayleigh scattering signals dominate the bright contrast of the image sensor. In this case, even if the Brillouin scattering spots were found, the strong background signal hinders us to estimate correctly the Brillouin shift and line width. In the latter, we can clearly see the Stokes and anti-Stokes Brillouin scattering signals of water (Wat.) and methanol (Met.) as well as Rayleigh scattering signals. The spots of the Rayleigh scattering (R.) appeared repeatedly in both directions are useful to calibrate the nonlinearity in converting the detection sensor number to the Brillouin shift.

Figure 4 shows the calibration procedure from the sensor pixel channel (ch) to the Brillouin shift (GHz). Figure 4a,b shows the sensor image of the Rayleigh and Brillouin scattering spot and the relation of the Rayleigh scattering spots (A, B, and C in Figure 4a) to the sensor pixel, respectively. The intervals between A and B and B and C correspond to 15 GHz. Since the intervals between A and B and those of B and C are different, we cannot assume the linear relation between the sensor pixel number and the Brillouin shift. Figure 4c shows the curve-fitting calibration with a quadratic function to correct the nonlinearity between the pixel number and the corresponding Brillouin shift. The quadratic function satisfactory converts the pixel number to the corresponding Brillouin shift. Figure 4d shows the calibrated Brillouin scattering spectra after the conversion of pixel number to the Brillouin shift. Utilizing three spots of Rayleigh scattering and not linear but nonlinear calibration with quadratic function enabled us to obtain less-distorted Brillouin scattering spectra.

**Figure 4:**
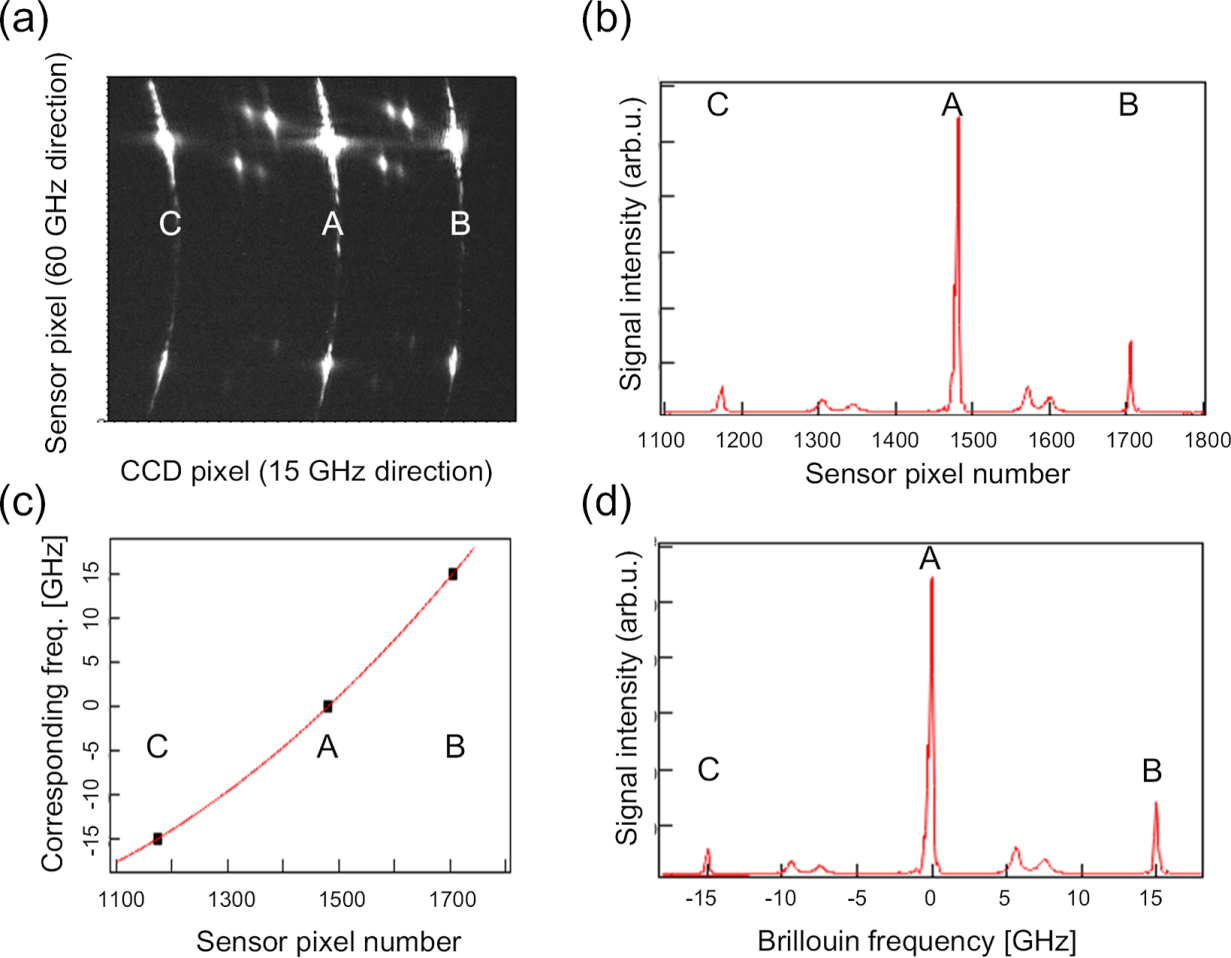
Procedure for the conversion of the pixel number of the sensor detector to the corresponding Brillouin shift. (a) Repeatedly observed spots of Rayleigh scattering (A, B, and C) in the 15 GHz FSR direction and Brillouin scattering signals between them. (b) Accumulation of the Rayleigh scattering and Brillouin scattering signals toward the 60 GHz FSR axis. (c) Conversion of the pixel number to the Brillouin shift by curve fitting with a quadratic function to correct the nonlinearity between the sensor pixel number and the corresponding Brillouin shift. Each interval between the Rayleigh scattering spots was 15 GHz. (d) The Brillouin spectrum with the frequency axis (GHz) estimated from the conversion from the sensor pixel number to the Brillouin shift shown in (c).

Then, the Brillouin shifts of the water in the optical cuvette in the sample arm and pure methanol in that of the reference arm were estimated. The procedure of the signal processing is shown in Figure 5 a–d. Stepwise curve fittings with Lorentz function to Brillouin scattering peaks and the removal of the background were successfully carried out. The estimated Brillouin shift and bandwidth of the 0 wt % BSA aqueous solution are 7.53 ± 0.090 and 0.801 ± 0.026 GHz, respectively. These values for the reference methanol solution are estimated from the curve fittings as 5.59 ± 0.039 and 0.531 ± 0.047 GHz, respectively. The slight differences between aqueous solutions with the presence and the absence of electrolytes, these values are in good accordance with those reported for water and methanol previously.^6,20–23^

**Figure 5:**
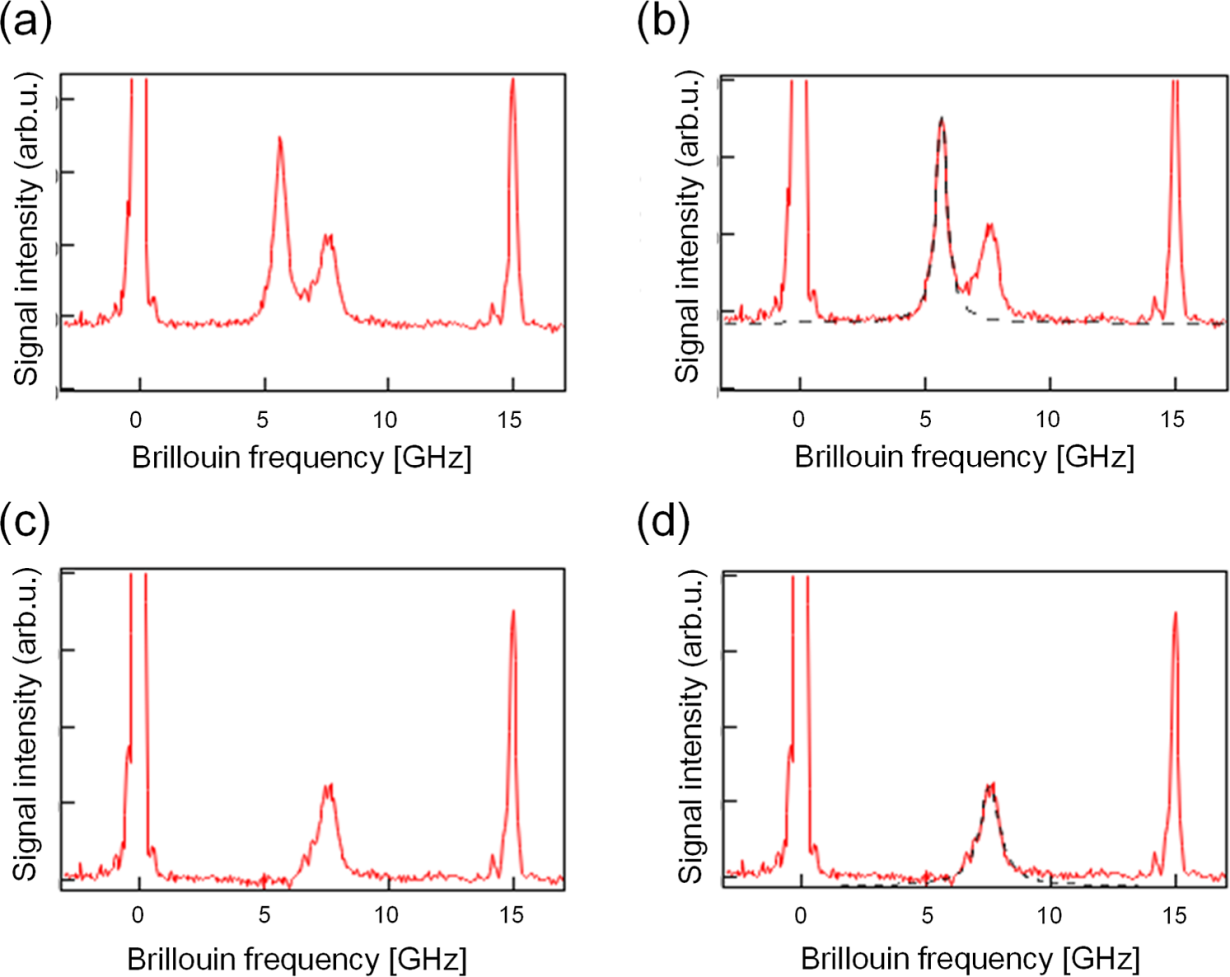
Estimation procedure for the Brillouin shifts and bandwidths of the sample (water) and the reference (pure methanol). (a) The raw spectrum of Brillouin scattering. We can see two Brillouin peaks between two strong Rayleigh peaks with the background. (b) At first, the Brillouin peak of the reference was fitted with a single Lorentz function with the background (dashed line). (c) Reconstructed spectrum after removing the reference and the background signals estimated in the procedure (b). (d) Then, we checked the symmetry of the remained sample peak to avoid the missing of the shoulder peak expected from the inhomogeneity of the sample solution. In the present case, the cuvette in the sample line is filled with pure water; a single Lorentz function was fitted to the remaining peak (dashed line).

Figure 6a,b shows the comparison of the simultaneously measured Brillouin shifts of the BSA aqueous solution (0–20 wt %) with electrolytes in the sample arm and those for pure methanol in the reference arm. In contrast to the stable frequency shift of pure methanol (at around 5.6 GHz), a monotonous increase in the Brillouin shift was observed from 7.57 to 8.13 GHz by increasing the BSA concentration. Figure 6c,d also shows the comparison of the simultaneously measured Brillouin bandwidths of the BSA aqueous solution (0–20 wt %) in the sample arm and those for pure methanol in the reference arm. Similar to the results shown in Figure 6a, the bandwidth of the methanol was stable during the measurements (Figure 6c) and that of the BSA aqueous solution showed a monotonous increase by elevating the BSA concentration (Figure 6d).

**Figure 6:**
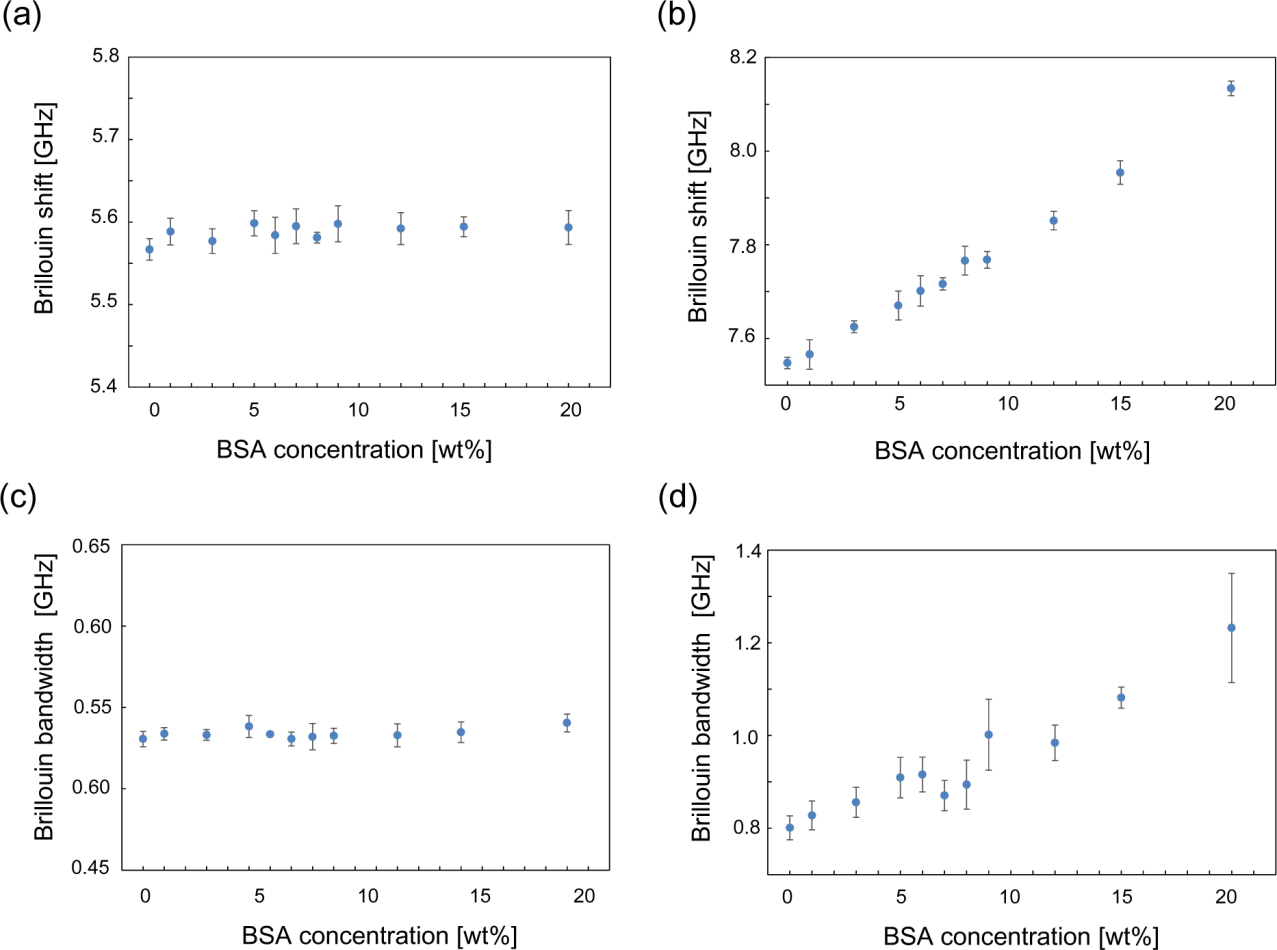
Results of the Brillouin shifts and line width of BSA solutions with varying BSA concentrations from 0 wt % to 20 wt %. (a) The monitoring of the fluctuation of the laser frequency with the reference methanol at the simultaneous measurement of the BSA solution. During the measurements, the stability of the laser frequency was confirmed by the simultaneously recorded reference signals. (b) The dependence of the Brillouin frequency of the BSA solution on its concentration. (c) The monitoring of the stability of the line width with the reference methanol at the simultaneous measurement of the BSA solution by varying its concentration. (d) The dependence of the Brillouin line width of the BSA solution on its concentration.

Figure 7a,b shows the curve-fitting analysis for the dependence of the Brillouin frequency and bandwidth on the BSA concentration (wt %). To check the stability of the laser frequency, we simultaneously monitored the Brillouin shift of the methanol for each measurement. The data are also listed in Table 1. During the measurements, the Brillouin frequency and the bandwidth of the reference methanol were kept almost constant, indicating the frequency of the laser was stable during the measurements when varying the BSA concentration.

**Figure 7:**
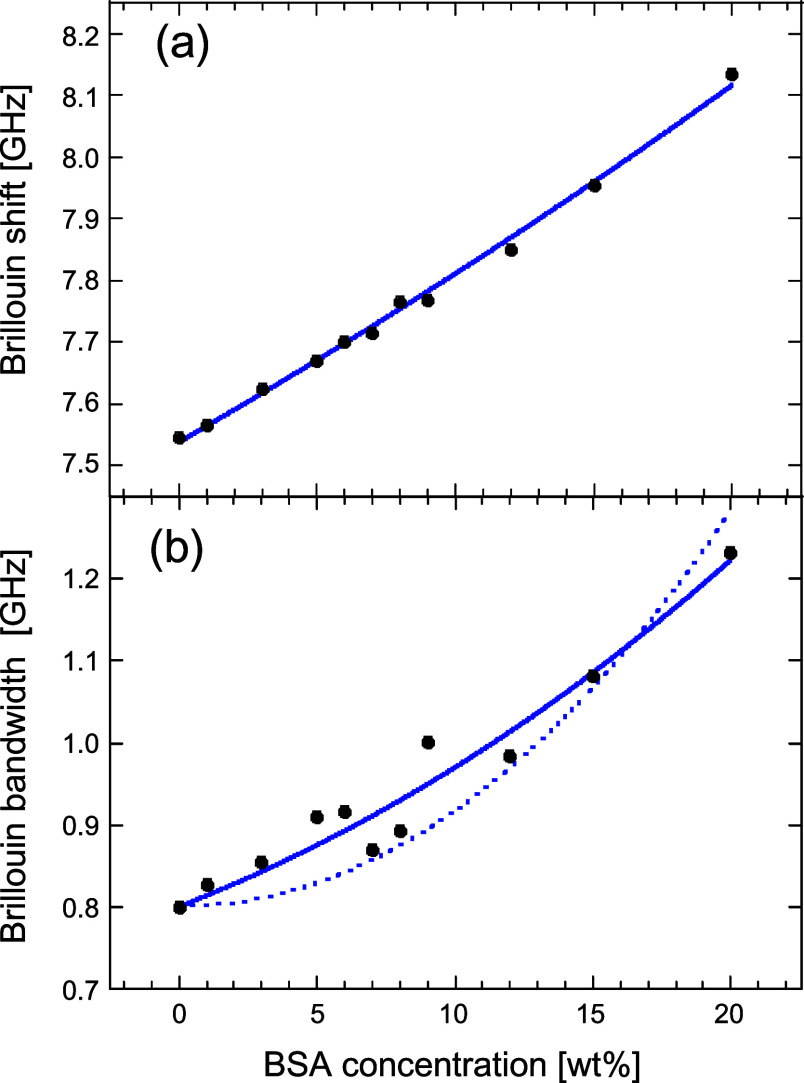
Dependence of the Brillouin shift and line width on the BSA concentration from 0 wt % to 20 wt %. (a) Curve-fitting analysis with eq 5 based on a biphasic model (solid line) described in the main text and (b) curve-fitting analyses based on the comparison between taking into account the hydration effect (solid line) and not (dotted line).

**Table 1:** The Dependence of the Brillouin Shift and Line Width on the BSA Concentration (wt %) and That of the Reference (Pure Methanol) Measured and Recorded Simultaneously

	Brillouin shift [GHz]		Brillouin bandwidth [GHz]	
BSA concentration	sample	reference	sample	reference
0	7.57 ± 0.015	5.56 ± 0.014	0.801 ± 0.026	0.531 ± 0.005
1	7.58 ± 0.026	5.59 ± 0.024	0.828 ± 0.031	0.534 ± 0.004
3	7.66 ± 0.007	5.60 ± 0.008	0.856 ± 0.032	0.533 ± 0.003
5	7.72 ± 0.009	5.60 ± 0.005	0.909 ± 0.044	0.538 ± 0.007
6	7.75 ± 0.021	5.62 ± 0.026	0.916 ± 0.038	0.533 ± 0.001
7	7.75 ± 0.022	5.62 ± 0.011	0.871 ± 0.053	0.531 ± 0.004
8	7.78 ± 0.004	5.63 ± 0.033	0.894 ± 0.077	0.532 ± 0.008
9	7.82 ± 0.010	5.59 ± 0.076	1.002 ± 0.038	0.533 ± 0.005
12	7.91 ± 0.029	5.62 ± 0.006	0.984 ± 0.023	0.533 ± 0.007
15	7.99 ± 0.023	5.60 ± 0.006	1.082 ± 0.023	0.535 ± 0.006
20	8.13 ± 0.020	5.61 ± 0.021	1.232 ± 0.118	0.540 ± 0.005

All data are the average of *N* = 4 measurements at each BSA concentration and the corresponding error is estimated by their standard deviation.

Figure 7a shows the dependence of the Brillouin frequency on the BSA aqueous solution. As mentioned above, pH and the electrolyte concentration (pH 7.4, Na^+^: 150 mM, K^+^: 5 mM, Ca^2+^: 1.2 mM, and Cl^–^: 157.4 mM) were adjusted to be similar to those in the lymph of human bodies. Previously, the concentration dependence of the BSA solution in pure D_2_O was reported.^24^ Although there were differences in experimental conditions between aqueous solution (H_2_O with electrolytes in the present study) and pure D_2_O, a similar increase in the Brillouin frequency with an increase in BSA concentration was observed.

The relation between Brillouin frequency ν_B_^eff^(*x*) and the mass fraction (*x*) of the solute (BSA) was successfully analyzed with the following equation based on a biphasic model typically for *x* < 0.4, where both the electrolyte aqueous solution phase and the hydrated biopolymer phase contribute to the speed of the acoustic wave propagation:

5 $$\nu_{B}^{eff}\left(x\right)=\frac{\nu_{B}^{W}}{\sqrt{1-x+x{\left(\frac{V_{W}}{V_{S}}\right)}^{2}}}$$

where ν_B_^W^ is the Brillouin frequency of water with the electrolyte (*x* = 0 wt %) and *V*_W_ and *V*_S_ are the sound velocities of the water phase and solute phase, respectively.^24^ The fitted curve with eq 5 is imposed to Figure 7a. Equation 5 excellently explains the frequency dependence of the Brillouin shift on the BSA concentration in our system. The best fitted values of ν_B_^W^ and (*V*_W_/*V*_S_) were 7.538 GHz and 0.997 [–], respectively. The former value shows good accordance with that obtained for water. The latter value suggests the sound velocity of the solute phase (i.e., BSA with hydration water shell) is quite similar to that of the electrolyte aqueous solution phase (Na^+^: 150 mM, K^+^: 1.5 mM, and Ca^2+^: 1.2 mW and Cl^–^: 157.4 mM) tested here. This result indicates that the BSA protein is well-hydrated and -dispersed. In addition, the electrolyte aqueous phase and the BSA solution phase are seamlessly connected from the viewpoint of density and elastic modulus according to the following relation:

6 $$V=\sqrt{\frac{M^{\prime }}{\rho }}$$

where *V* is the acoustic velocity, ρ is the medium density, and *M*′ is the real part of the complex longitudinal modulus. On the other hand, the imaginary part of the complex longitudinal modulus (*M*″) provides information on the acoustic attenuation, which can be related to the Brillouin bandwidth.^5^

Then let us discuss the dependence of Brillouin bandwidth (Γ_B_(ν_B_^eff^) GHz, fwhm) on the BSA concentration (*x* wt %). The Brillouin bandwidth can be related to the apparent viscosity η (ν_B_^eff^) by the following relation:^25^

7 $$\eta \left(\nu_{B}^{eff}\right)\propto \Gamma_{B}\left(\nu_{B}^{eff}\right)/\nu_{B}^{eff}{\left(x\right)}^{2}$$

At the relatively low BSA concentration (*x* < 0.20, in human bodies *x* < 0.10), eq 7 can be simplified by the proportionality coefficient *A* and as a function of *x* as follows:

8 $$\Gamma_{B}\left(\nu_{B}^{eff}\right)=A\nu_{B}^{eff}{\left(x\right)}^{2}$$

At first, we fitted eq 8 to the experimental results of Γ_B_(ν_B_^eff^) (Figure 7b). However, eq 8 does not well fit the results, especially in lower concentration of BSA (*x* < 0.1). Another probable contribution to the broadening of the Brillouin bandwidth is the relaxation of the hydrated water molecules surrounding the biological polymers, i.e., BSA.^25^ When the hydrated water molecules increase, a proportional contribution to the Brillouin bandwidth via the water relaxation susceptibility takes place below 90 GHz.^25^ To take the proportional increase of the hydrated water molecules to BSA protein concentration (*x*) into account, we extended eq 8 to eq 9 by adding proportionality coefficient B as follows:

9 $$\Gamma_{B}\left(\nu_{B}^{eff}\right)=A\nu_{B}^{eff}{\left(x\right)}^{2}+Bx$$

Eq 9 well-explains the experimental data (*A* = 0.014 and *B* = 0.854) in the most relevant range (*x* < 0.1) for the application to the living bodies. The remarkable contribution of the hydrated water term indicates that even under the relatively lower concentration of protein aqueous solution (*x* < 0.2), the range and the quantities of hydrated water molecules to the proteins induce non-negligible contribution to the attenuation of the sound propagations via the friction to the surrounding media of proteins. In another word, we should take the effect of the hydrated water molecules of proteins into account to discuss the mechanical properties of the protein aqueous solution. In addition, it should be noted that the remarkable contribution of the number of hydrated water molecules also supports the experimental results shown in Figure 7a, where the *V*_W_/*V*_S_ was almost 1, indicating that the solute phase includes many water molecules by hydration. If the concentration of the solute molecules exceeds 0.2 (*x* > 0.2, above the present concentration conditions), the contribution of solute–solute interaction (i.e., crowding effect between polymer segments) to the viscosity of the medium should be also taken into account.^25^

## Conclusion

The effective reduction of strong background signals that often hinder the Brillouin scattering measurements was demonstrated by applying off-axis illumination and epicollection geometry for confocal Brillouin microspectroscopy. Since typical tissues have three-dimensionally inhomogeneous structures and biorelevant fluids such as blood and lymph flow in these tissues, the proposed optical configuration would provide an effective option to address the viscoelastic properties of such buried tissues and biological fluids therein. The reference optical line is also constructed to monitor and record the Brillouin shift and line width of the reference sample. Simultaneous monitoring and recording of the reference would contribute to the robustness of the μCBS to unexpected fluctuation of the temperature in the laser system for a long-time operation and allow us to calibrate the Brillouin shift and line width of the sample afterward. The dependence of the Brillouin frequency shift and line width to the BSA concentration in its aqueous solutions with biologically relevant electrolytes was successfully analyzed by considering the contribution of the hydrated water molecules surrounding BSA molecules. The proposed analytical technique would provide a useful microscopic tool for biomedical diagnosis via the local differences of the viscoelastic properties in the buried position. In addition, gradual changes of viscoelastic properties occur on the time scale of hours in biorelevant fluids such as blood and lymph due to the changes in the number and structures of proteins and surrounding hydrated water molecules induced by biochemical reactions with nonlabeling and noninvasive manners for biochemical analysis.
